# Fish remains as a source to reconstruct long-term changes of fish communities in the Austrian and Hungarian Danube

**DOI:** 10.1007/s00027-015-0393-8

**Published:** 2015-06-03

**Authors:** Alfred Galik, Gertrud Haidvogl, Laszlo Bartosiewicz, Gabor Guti, Mathias Jungwirth

**Affiliations:** Institute of Anatomy, Histology and Embryology, University of Veterinary Medicine Vienna, Veterinärplatz 1, 1210 Vienna, Austria; Institute of Hydrobiology and Aquatic Ecosystem Management, University of Natural Resources and Life Sciences, Vienna, Austria; Institute of Archaeological Sciences, Eötvös Loránd University, Budapest, Hungary; School of History, Classics and Archaeology, University of Edinburgh, Edinburgh, Ireland; Danube Research Institute, MTA Centre for Ecological Research, Hungarian Academy of Sciences, Budapest, Hungary

**Keywords:** Archaeoichthyology, Archaeological methods, Fish community change, Austrian Danube, Hungarian Danube

## Abstract

The main objective of this paper is to investigate how archaeological fish remains and written historical records can contribute to the reconstruction of long-term developments of fish communities along the Austrian and Hungarian Danube. Although such approaches are sensitive to various factors, the chronological subdivision and relative quantification of proxy data demonstrate environmental and faunal changes from Prehistory onwards. Intensification of fisheries, decline of large specimens and massive exploitation of small and young fish point to increasing pressure along the chronological sequence towards Early Modern times. One result of this impact was the establishment of regulations and laws to protect such fish. At the same time, the rise of aquaculture and common carp cultivation can be viewed as another upshot of human impact on the Danube’s environment. Finally, the massive import of salted marine fish reflects a compensation for the undersupply caused by overexploitation of the Danube fish fauna and points to the growing demand for fish as food in late medieval and Early Modern times.

## Introduction

The Danube comprises a multitude of aquatic environments along its course from the German Schwarzwald to Austria, Hungary and further south into the Carpathian Basin. Although the river endangered riparian communities by recurring flooding, humans also exploited the natural habitats along the Danube as documented by numerous archaeological sites along the Austrian and the Hungarian part (river km 2135–1886 and 1796–1581). Excavations here uncovered refuse deposits containing fish remains and prove that fishing has substantially contributed to human nutrition since the Neolithic (Bökönyi 1974; Radu 2003; Bartosiewicz 2013).

The successful identification of many fish bones to species level is certainly based on the availability of reference collections. The investigations of such archaeological remains as proxy data address many societal and ecological questions. Cultural preferences influence various social levels as comparing monastic and aristocratic households or civil sites including latrines and waste disposal places (Galik 1999). Many factors weaken the signals of such proxies, for example their unknown taphonomic history, their inhomogeneity or the precision of recovery. Fish remains from sieved samples significantly differ from hand-collected samples, which yield only few but large specimens (Jones 1983; Bartosiewicz 1988; Cao et al. 2002; Gobalet 2005; Zohar and Belmaker 2005; Chao et al. 2009). Therefore, screening of sieved sediment samples is a precondition for the reliable recovery of fish remains, independent of diverse taphonomic potentials of preservation in various contexts. Especially latrines offer extraordinary conditions as cavities in which masses of material were concentrated during short time spans and excellent preservation conditions conserved even fragile and tiny fish bones (Heinrich 1995; Brombacher et al. 1998; Nussbaumer and Rehazek 2007).

Ecological approaches usually relate to manmade faunal changes or impacts on ecosystems (Kurlansky 1999; Jackson et al. 2001; Pitcher 2001) and, in the case of fishes, environmental pressure can be illustrated by the size patterns of reconstructed lengths of specific species (Jackson et al. 2001). Nonetheless, information derived from archaeological proxy data cannot be simply transformed into measureable variables. Rather, according to Pauly (1995), they are more like “anecdotes”. According to Jackson et al. (2001) “the precision and clarity of the signal they measure” appears to be somewhat unclear but a framework combining historical and archaeoichthyological data as proxies produces stronger signals on a long-term scale. Nevertheless, the finds along the Austrian and Hungarian part of the Danube clearly bear the potential to detect changes chronologically and at a regional scale in a riverine milieu providing new societal and environmental data beyond written documents (Haidvogl et al. 2014). Using such information about past ecological conditions raises a crucial question related to the provenance of the fish. Especially for the later periods, it often remains uncertain if the archaeological fish remains originated from local waters or if they were traded from distant places. For continental sites such as the upper and middle Danube, this is easy to answer only in the case of marine fish.

The main objective of this paper is to test the capacity of such remains to contribute to reconstructing past fish ecological conditions. This involves (1) analysing the influence of recovery methods on the resulting species list, (2) investigating whether the relative abundance of fish species differs on large spatial and temporal scales, (3) identifying size distributions and (4) comparing the fish remains from the early modern period with written documents to reveal differences between two distinct types of proxy information.

## Materials and methods

### Provenance of the archaeological fish remains

**Fig. 1:**
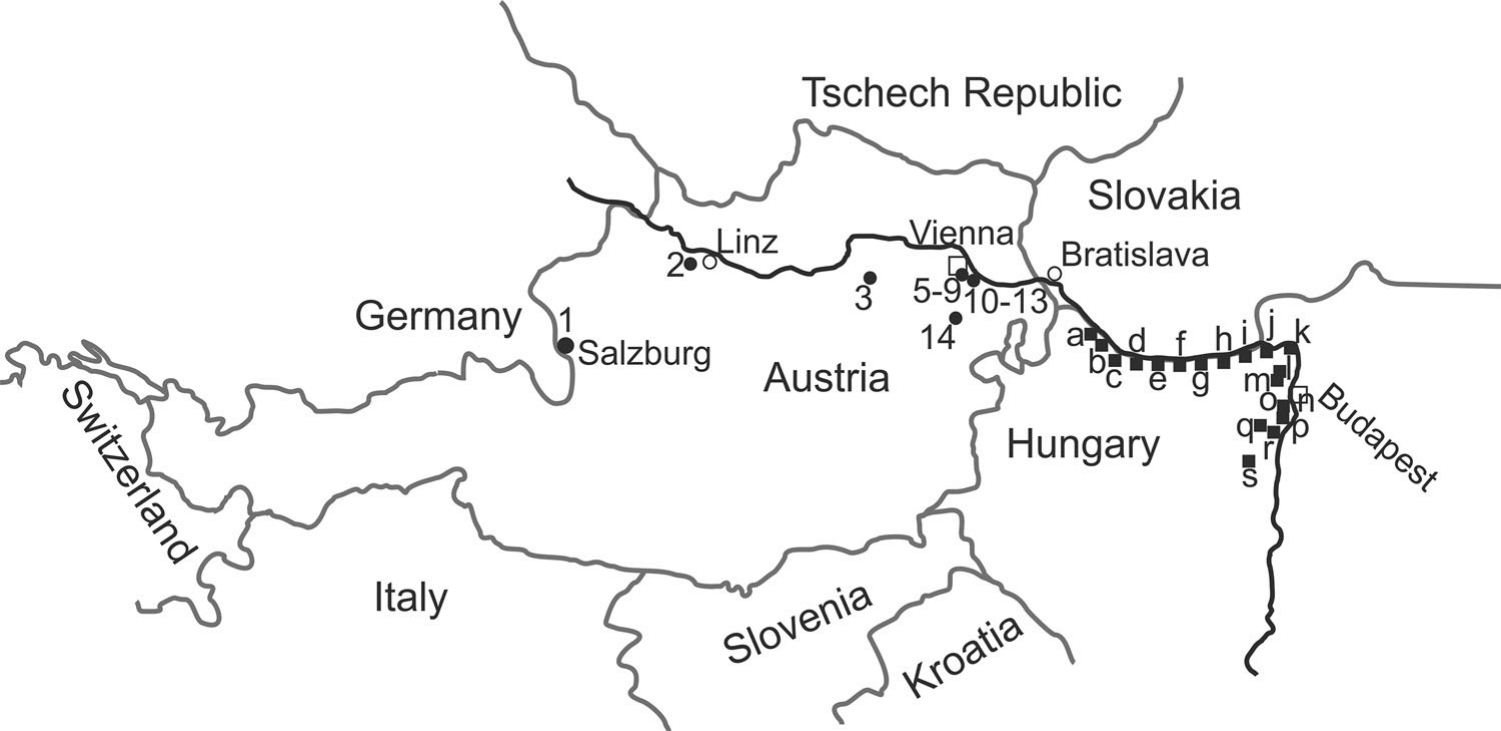
Map with the Austrian (*1–14*) and Hungarian sites (*a–s*) along the Danube and tributaries (line drawing Galik). Data: *1* Pucher (1991); *2* Galik (2008); *3* Galik et al. (2011); *4* Galik (1998), Galik and Kunst (2000), Kunst and Galik (2000); *5*–*13* data for Vienna: Galik unpubl, Jandl and Mosser (2008), Mosser (2010); data for Carnuntum: Galik unpubl, Galik et al. (2009), Petznek (2012); *14* Galik unpubl, Kühtreiber (2005, 2006). *a* Bartosiewicz unpubl., *b* Bartosiewicz et al. (1994), *c* Bartosiewicz unpubl., *d* Bartosiewicz (1989), *e* Bökönyi (1974), *f* Bökönyi (1974), *g* Bökönyi (1974), *h* Takács unpubl, *i* Bökönyi (1974), *j* Bartosiewicz unpubl.; *k* Bartosiewicz (1995), *l* Bökönyi (1974), *m* Takács-Bartosiewicz unpubl., Takács unpubl., *n*, *o* Bartosiewicz (2003), Bökönyi (1963), Bökönyi (1958), Takács and Bartosiewicz unpubl., Takács unpubl., Bökönyi (1959), *p*, *q* Bökönyi (1974), Bökönyi (1959), *r* Bökönyi (1974), *s* Bökönyi (1959)

The archaeoichthyological material comes from sites along the Danube in Austria and Hungary. The Austrian river section is characterized by alpine influences in terms of velocity and temperature. Downstream of Bratislava and Györ the character shifts towards a lowland river, also reflected by a change from a dominance of rheophilic and eurytopic to more limnophilic cyprinids (Schiemer et al. 2004). The Austrian Roman, medieval and post medieval sites are concentrated in the Vienna Basin (Fig. 1: 5–13), which showed—prior to the channelization in the 19th century—the typical pattern of an anastomosing river system. This basin was inhabited by diverse fish communities comprising about 52 native species including the sturgeon species (Schiemer and Waidbacher 1998; Spindler 1997). Austrian fish data include archaeological sites at tributaries, among them a post-medieval cesspit in Salzburg near the Salzach River associated with a tavern (Fig. 1: 1). Ansfelden at the confluence of the Traun and Krems rivers documents a long history of settling activities starting in the Neolithic and terminating after early medieval times (Fig. 1: 2). A late medieval monastic latrine filling in St. Pölten is located close to the Traisen River (Fig. 1: 3). Another post-medieval site, the Carthusian monastery Mauerbach was situated at a brook of the same name; that brook was no doubt too small to sustain fish stocks large enough to supply a larger number of friars (Fig. 1: 4). Foundations of the medieval castle Dunkelstein near the Schwarza River, which discharges into the Leitha River and finally ends in the Danube, also revealed fish remains (Fig. 1: 14). In total, approximately 16,000 fish remains were recovered from the Austrian sites.

The Hungarian sites are situated at the upper part of the Hungarian Danube, approximately halved by the Danube Bend gorge where it takes a sharp turn toward the south. The long course is differentiated in an “upstream” and a “downstream” section. The upstream section of the Hungarian Danube begins at the eastern tip of the Szigetköz region. The sites Börcs-Paphomlok-dűlő (Fig. 1: a), Győr-Szabadrét-domb (Fig. 1: b) and Ménfőcsanak-Széles telep (Fig. 1: c) are situated upstream the hilly Danube Bend gorge near Visegrád. The river flanked by floodplains becomes gradually restricted and offered space for sites in Ács-Vaspusztal (Fig. 1: d), Neszmély-Tekeres (Fig. 1: e), Süttő-Hosszúvölgy (Fig. 1: f), Nyergesújfalu-Téglagyár (Fig. 1: g), Esztergom-Királyi (Fig. 1: h), Pilismarót-I. őrtorony (Fig. 1: i) and finally several sites at Visegrád (Fig. 1: j) and downstream at Vác (Fig. 1: k).

The downstream section of the Danube starts after the southward turn. Sites of various chronological stages are at Békásmegyer (Fig. 1: l) and Óbuda (Fig. 1: m). The river flows to a plain near Budapest and becomes wider and less oxygenated because the floodplain area is significantly broader; numerous sites are located at Buda-Vár (Fig. 1: n, o), in Budapest itself (Fig. 1: p, q) and at Csepel-Háros (Fig. 1: r). To date no archaeological fish bone assemblages are available from Hungary further downstream from the southernmost site Dunapentele (Fig. 1: s). The 52 Hungarian sites reviewed in this study provided approximately 2000 specimens.

### Identification and aggregation of data

The archaeoichthyological material was identified using ichthyo-osteological reference collections. Measurements on various skeletal elements were taken according to Morales and Rosenlund (1979), and size reconstructions for cyprinids were calculated according to Radu (2003), Radke et al. (2000), Desse et al. (1989) and Desse and Desse-Berset (2005).

Screening of sieved sediment samples was employed only at the Austrian sites, i.e. the castle Dunkelstein, the mainly prehistoric settlements in Ansfelden as well as the latrine fillings from Petronell, St. Pölten and the Stallburg in Vienna, where most of the fish bones come from. In order to identify the influence of the sampling effort on reconstructed fish species, we correlated the number of identified bones vs. the number of identified species.

The abundance of identified fish species is a descriptor of particular fish assemblages which indicate different ecological river types. Its reliability does largely depend on high numbers of skeletal remains within the archaeoichthyological samples. However, sample sizes are highly variable in the sites along the Danube, some providing numerous specimens and others only a few finds. Nevertheless, such comparisons can indicate certain ichthyological- (Lepiksaar 2001) and historical developments (Makowiecki 2000). Makowiecki (2003) thoroughly discussed the Polish lowland ichthyofauna based on the archaeological frequencies of species with regards to possible distortion arising from varying numbers of skeletal remains. The specific compositions of species in relation to climatic and chronological periodization including size reconstruction of fishes clearly produced pattern induced by the surrounding habitat of the sites from the Mesolithic towards the medieval period and the Modern Times (Makowiecki 2003). Therefore, the use of such a comparative technique promises to reconstruct ecosystem changes following the diachronic sequences along the Austrian and Hungarian parts of the Danube, although faunal peculiarities might be driven by local and regional developments as well.

Four main units were considered: prehistoric, Roman, medieval, late/post-medieval. The medieval period covers a few finds from the early 9th century, while the major part represents the high medieval era up to the 13th century. The late/post-medieval sequence summarizes the late medieval epoch, with remains from the 14th/15th centuries and Early Modern Times with finds from the 16th/17th centuries. Sites along the Danube in the Vienna Basin were combined as one study site, and sites along different tributaries were evaluated as a second spatial unit in Austria. In Hungary two main units—the “upstream” section towards Vác and the “downstream section” downstream the bend gorge—were used based on the definition of hydro-morphological units for the Joint Danube Survey (Sommerhäuser et al. 2003). Identified cyprinids were compiled according to their degree of rheophily (Noble et al. 2007).

Finally, the archaeological fish assemblages were compared with two types of written historical sources. For Austria, we used a description of fish species offered for sale at the Viennese fish market in 1540 (Schmelzl 1547; Tab. 1) and for Hungary we took into account the cookbooks from Galgóczi in 1622 (Herman 1887; Tab. 1). Such descriptions for an urban market and for human consumption are subject to societal filters that differ from those of archaeological fish remains. Importantly, they are not affected by taphonomic processes.

## Results

### Sampling effort

**Fig. 2:**
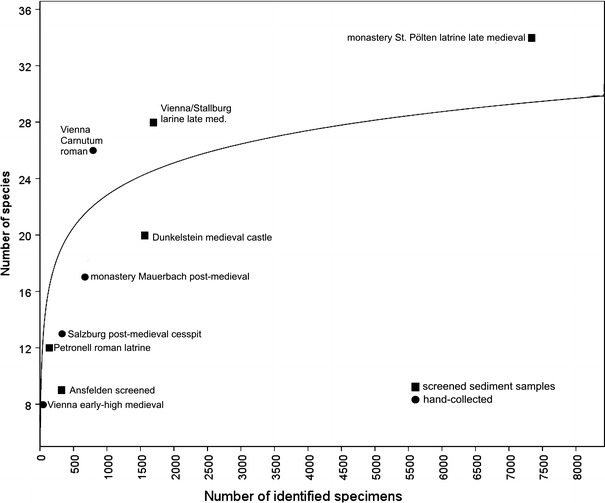
Relation of sample size and richness of species from sites including hand collection and screened sieved sediment samples (*y* = 1 × exp(0.3026 × *x*))

Evaluation of hand-collected and sieved sediment samples supported the conclusion that sampling effort, i.e. number of bones collected and identified, influences the species richness, although outliers exist (Fig. 2). Remains from sieved samples from the two late medieval latrines yielded the highest frequencies and species numbers. The latrine in Vienna yielded 28 and the latrine in St. Pölten 34 species. The Roman latrine did not produce high quantities of specimens or species, whereas the carefully hand-collected Roman sites in the Vienna Basin (Carnuntum) exposed a high diversity of species but a low number of identified specimens. The sieved sediment samples from tributaries at Ansfelden and at the castle Dunkelstein yielded fish remains with a lower species number. Most hand-collected Austrian and Hungarian sites clearly indicated low frequencies of species and specimens. They usually represented large remains of sizeable species, while screening of sieve residues recovered all kinds of fish bones including remains of small species.

### Relative abundance of identified species

**Fig. 3:**
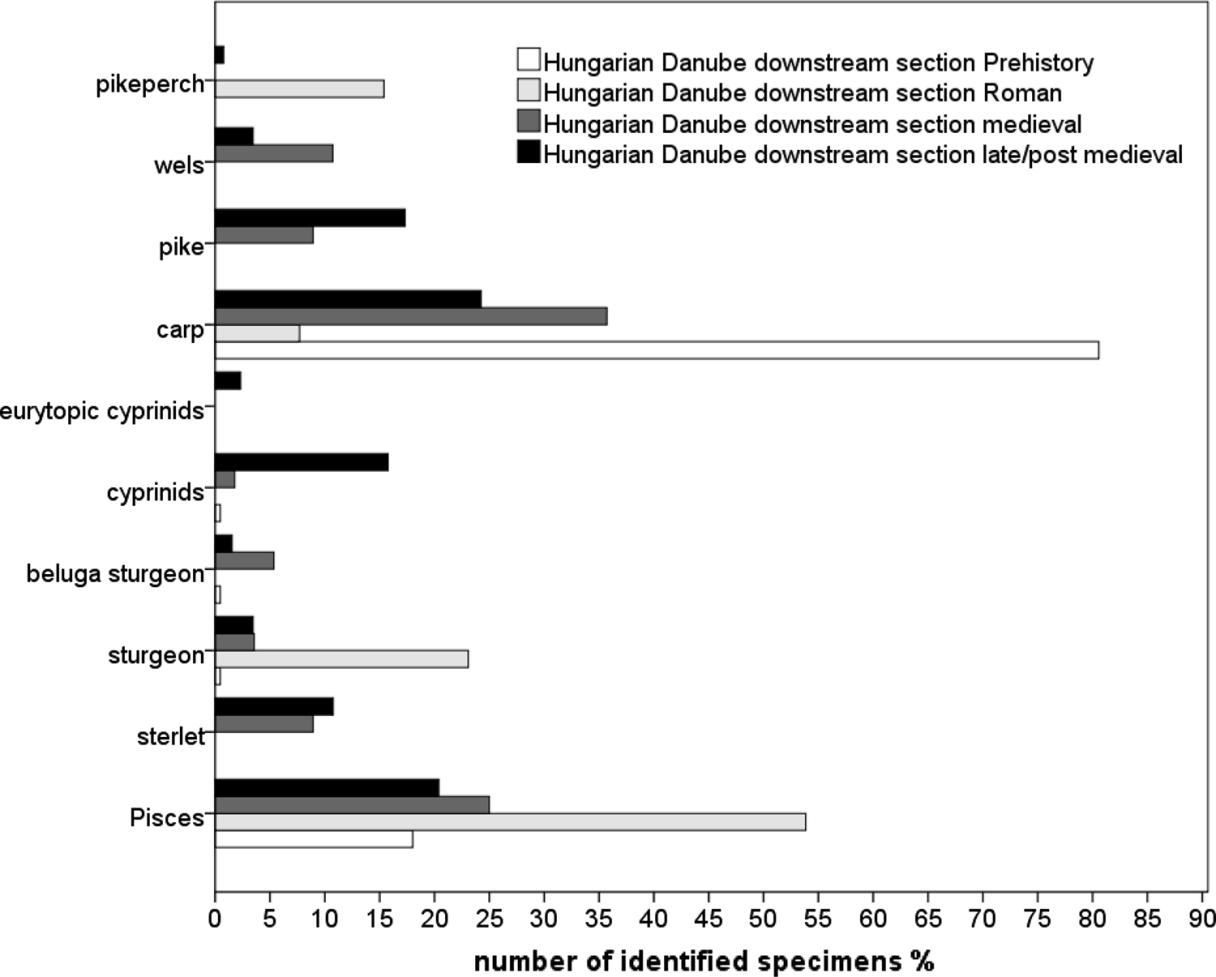
Relative abundance of fish from the downstream section of the Danube in Hungary (Pisces include all unidentifiable fish bones from an archaeological sample)

**Fig. 4:**
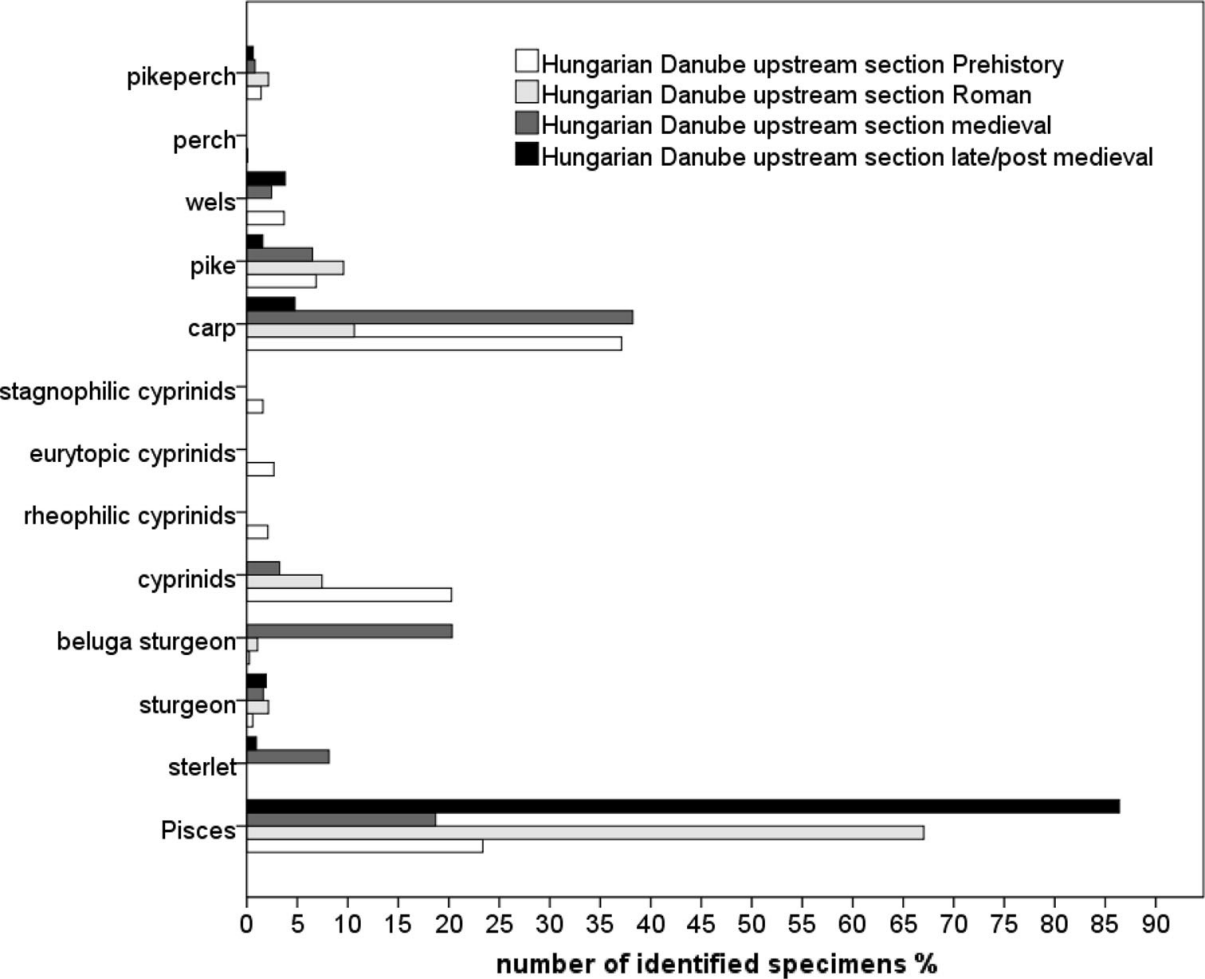
Relative abundance of fish from the upstream section of the Danube in Hungary

**Fig. 5:**
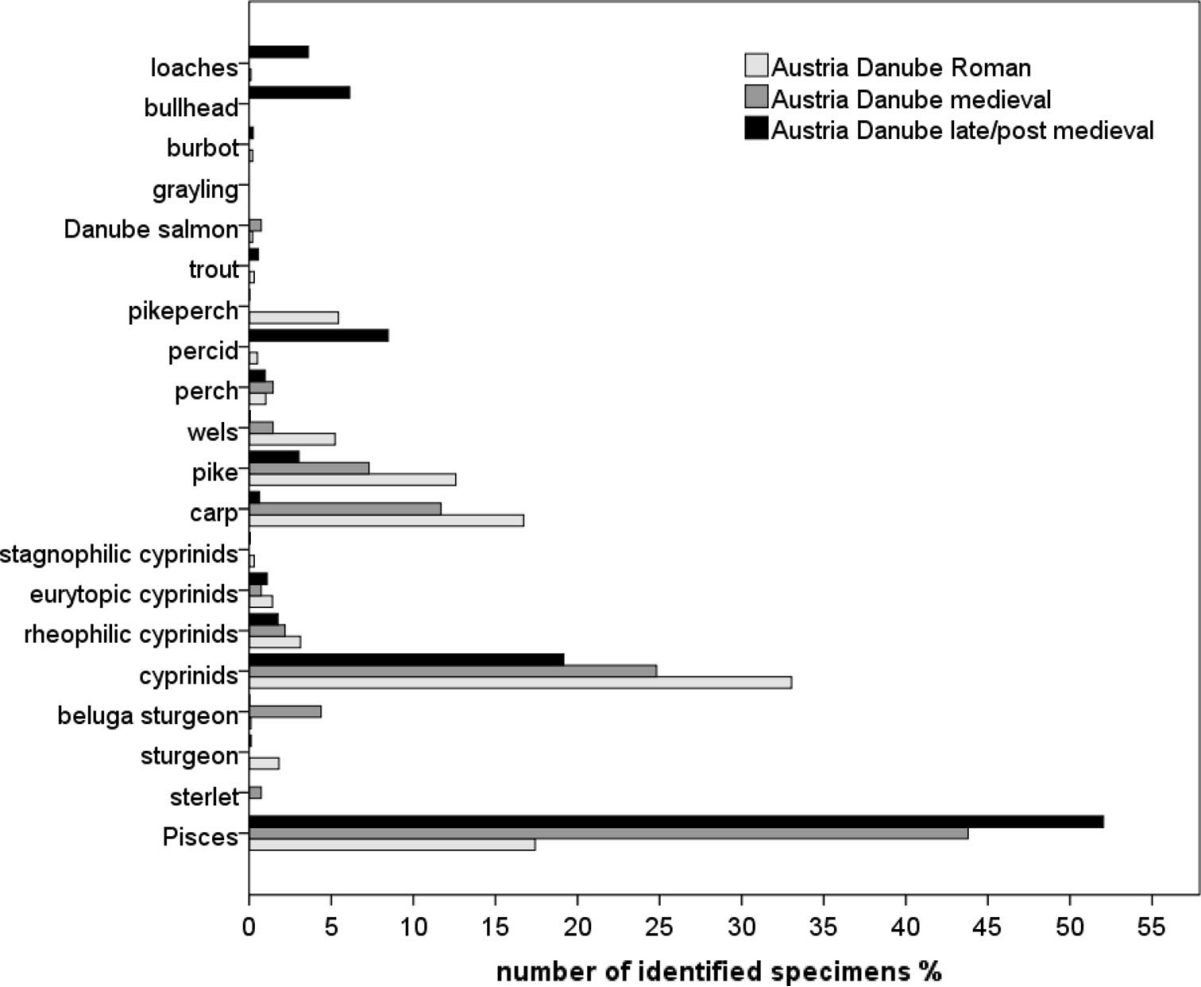
Relative abundance of fish from the Danube in the Vienna Basin

**Fig. 6:**
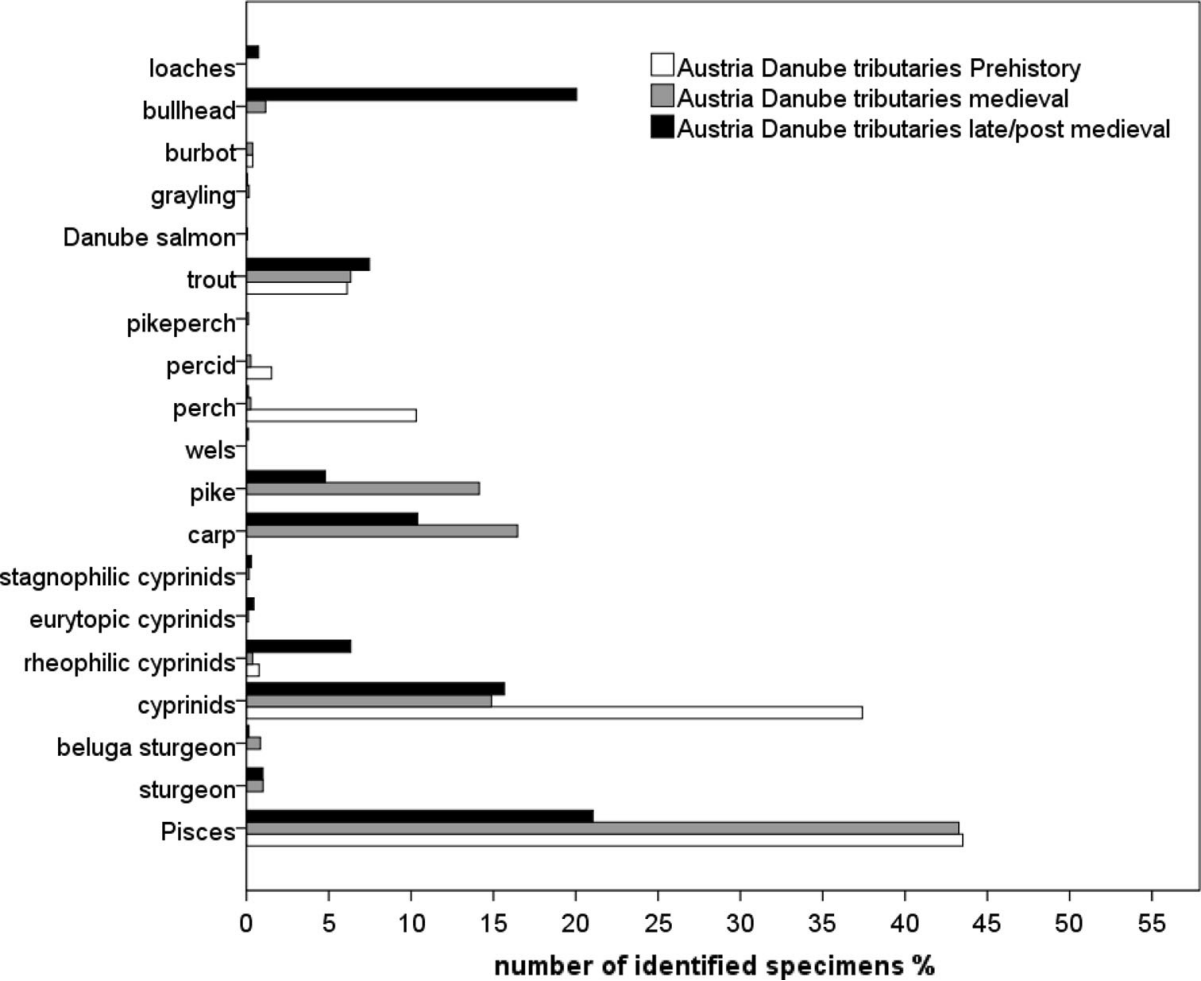
Relative abundance of fish from the Danube tributaries in Austria

A few prehistoric sturgeons were recovered in the Hungarian sites. While missing at Austria’s prehistoric site at tributaries, sturgeons were present in Roman sites along the Danube (Figs. 3, 4, 5, 6). The later periods proved Danube sturgeon to be present not only in the Danube but also in sites at the tributaries, although quantitatively they were rare in the late/post-medieval periods. Both sections of the Hungarian Danube reflected a higher abundance especially in the late/post-medieval, with sterlet being the main species in the downstream section (Figs. 3, 4).

All sites revealed highly abundant unidentifiable fish- and unidentifiable cyprinid remains, and only a few rheophilic, eurytopic and limnophilic species were identified in the Hungarian sites (Table 1). Eurytopic cyprinids constitute the highest proportion of cyprinids, except in prehistoric times in the upstream section of the Hungarian Danube (Table 1). All periods in the Vienna Basin and especially at the tributaries contained abundant rheophilic cyprinids, fewer eurytopic and only a few stagnophilic cyprinids.

Common carp (*Cyprinus carpio*) was overwhelmingly represented in the prehistoric contexts at both sections of the Hungarian Danube but it remained absent in Austria’s prehistoric site at the Danube tributaries. The Roman-, medieval- and late/post-medieval contexts revealed similar patterns at least for the Vienna Basin and the upstream section of the Hungarian Danube. Although common carp appears quite opposite in the Roman contexts, the decline in abundance from medieval- to late/post-medieval contexts reveal similar pattern at the Hungarian and Austrian sites. The frequencies of pike are similar in Roman contexts as well as the decrease in abundance towards late/post-medieval sites in the Austrian sites and sites at the upstream section of the Hungarian Danube (Figs. 3, 4, 5, 6). The downstream section of the Danube in Hungary yielded no prehistoric or Roman pike remains and showed an increase of pike towards late/post-medieval periods (Fig. 3).

Most of the Austrian and few Hungarian sites yielded perch (*Perca fluviatilis*; Tab. 2). The late medieval latrine in the Vienna Basin contained schraetzer (*Gymnocephalus schraetzer*) and ruffe (*Gymnocephalus cernua*/*baloni*). Pikeperch (*Sander lucioperca*) was particularly well represented in the Roman contexts at the Austrian and Hungarian Danube but appeared in smaller proportions in later periods and at the Austrian tributaries (Figs. 3, 4, 5, 6).

Wels (*Silurus glanis*) was clearly a regular catch around the Danube, but it remains rare at the Austrian tributaries. Its highest frequency in Austria occurred in the Roman period (Figs. 5, 6). The upstream and downstream sections of the Hungarian Danube indicate a higher abundance in medieval and late/post-medieval times (Figs. 3, 4).

Brown trout (*Salmo truttaf. fario*) occurred in high proportions in all chronological periods at the Danube tributaries in Austria, along with rare evidence of Danube salmon (*Hucho hucho*) and grayling (*Thymallus thymallus*, Table 1). The Danube in the Vienna Basin yielded less brown trout from Roman and late/post-medieval sites but higher frequencies of Danube salmon in Roman and medieval periods. A few finds of burbot (*Lota lota*) imply occasional catches at Austrian sites. Salmonids as well as burbot did not occur in any Hungarian site.

Loaches (*Barbatula* sp., *Cobitis* sp.) were present in the Austrian late medieval- as well as in the Roman latrine. Bullhead (*Cottus gobio*) was documented in the sites related to the Danube and along the tributaries, for example at the medieval castle. Although small fish were present in Roman and medieval times, masses accumulated in the late medieval context only.

**Fig. 7:**
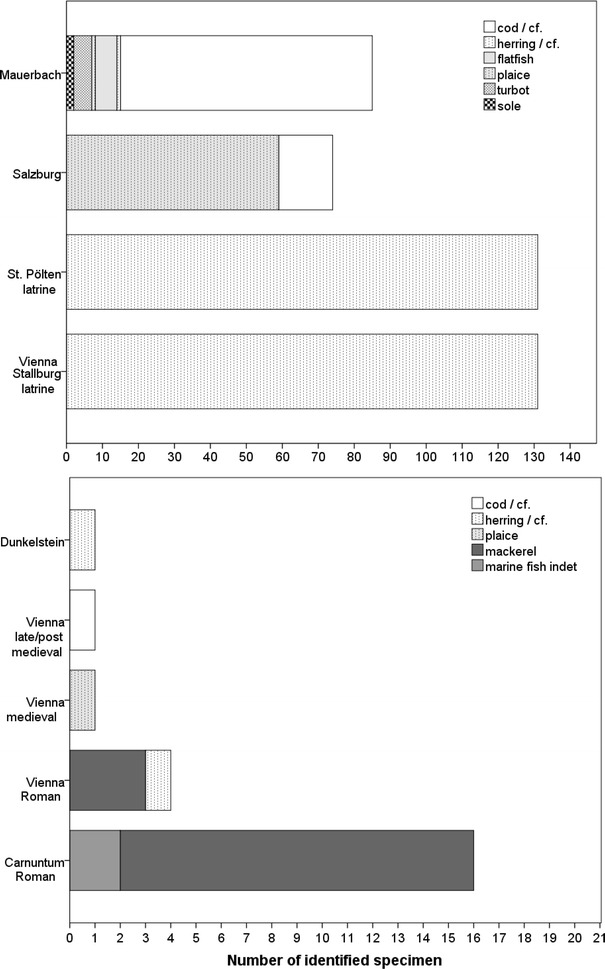
Abundance of marine fish remains in Austria (*diagram above lists* Austrian sites with numerous remains of marine fish, *diagram below lists* sites with rare finds of marine fish)

Besides above-described species native in the Danube, catadromous eel (*Anguilla anguilla*) occurred in the late medieval latrines in the Vienna Basin and in St. Pölten. Other remains demonstrated the import of marine fish back as far as the reign of the *Imperium Romanum*. The most abundant species was Atlantic chubb mackerel (*Scomber scolias*). While the medieval castle yielded only a single specimen, the latrines produced large numbers of herring vertebrae. Other imported species included cod as stock fish (*Gadus morhua*) and flatfish, mainly plaice (*Pleuronectes platessa*) but also sole (*Solea solea*) and turbot (*Scophthalmus maximus*) in monastic contexts and in the cesspit filling of the tavern in Salzburg (Fig. 7; Table 1). Contrary to the Austrian evidence, so far no Hungarian sites have yielded marine fish remains.

### Changes in size distribution of cyprinids and common carp

**Fig. 8:**
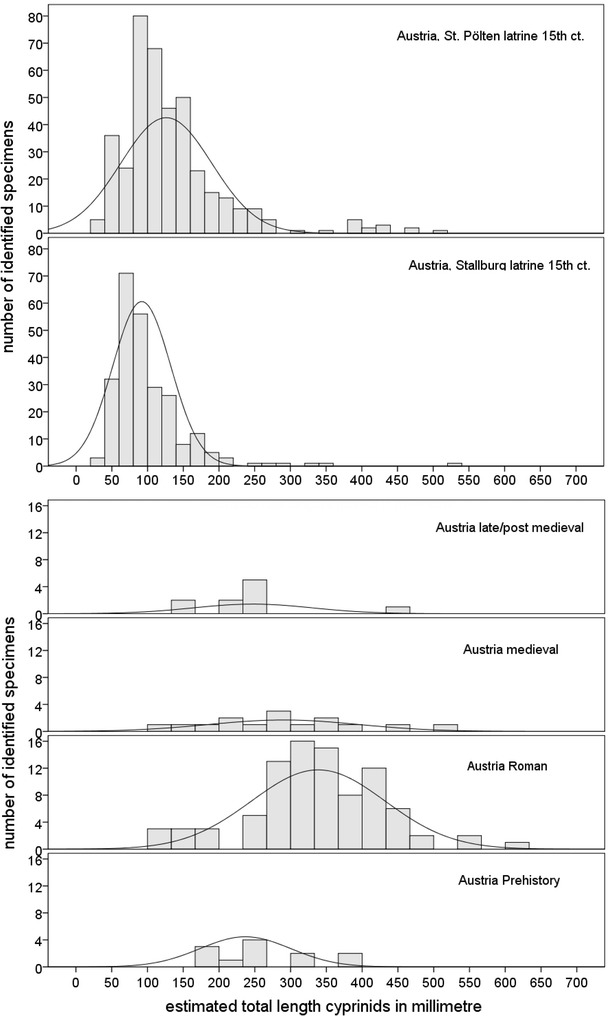
Estimated size distribution of cyprinids from Austrian sites (*diagram above* indicates the size distribution of cyprinids from late medieval latrine fillings in Vienna—Stallburg and St. Pölten,* diagram below* indicates the size distribution of cyprinids from prehistoric to late/postmedieval sites and only the Roman assemblage contains remains form a latrine filling)

**Fig. 9:**
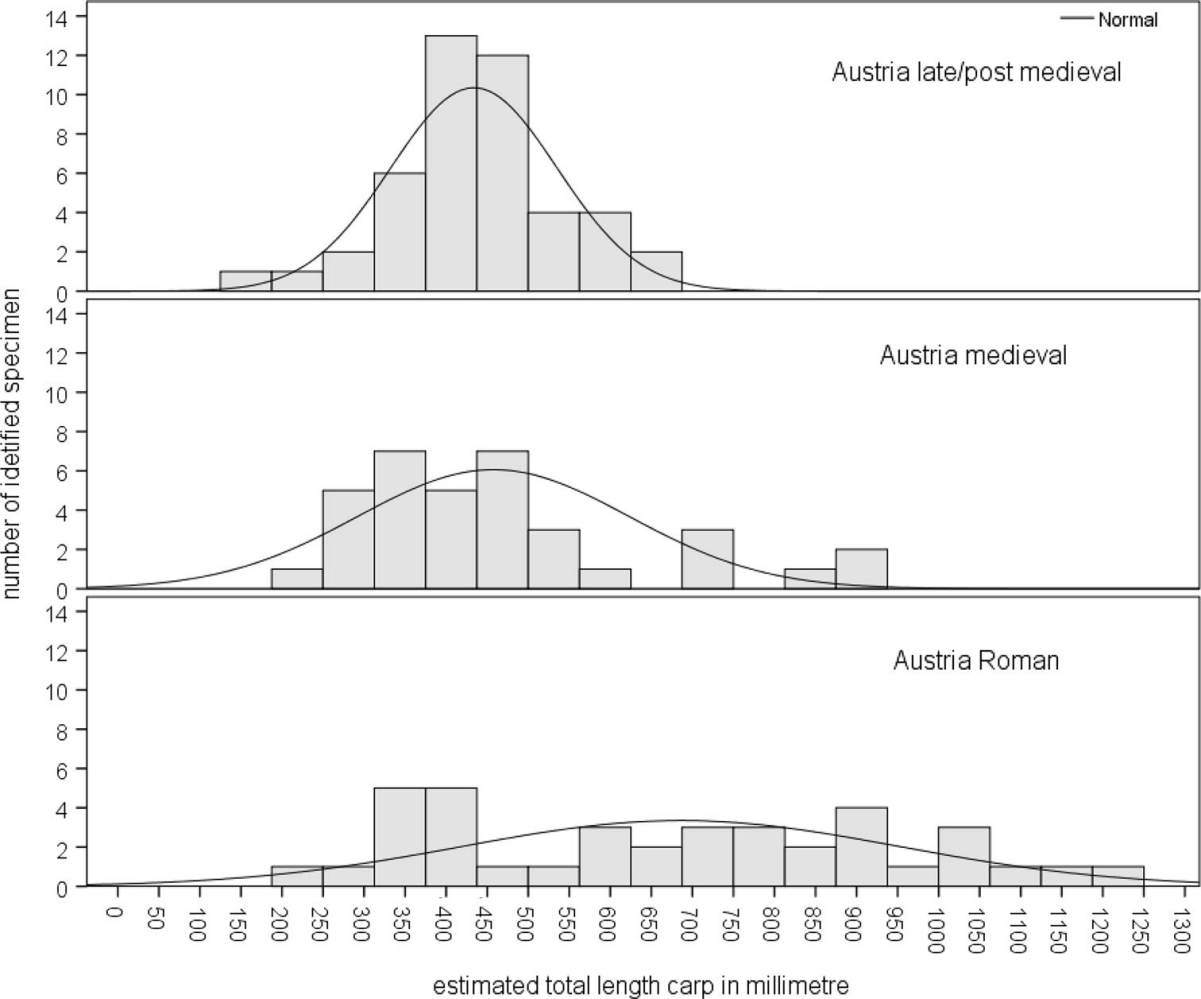
Estimated size distribution of common carp at sites in Austria

Most of the material indicated cyprinids in the size range of 30–40 cm and sometimes even larger (Fig. 8). The Roman latrine yielded only few data, revealing small fish measuring only about 10 cm (Fig. 8 below). The late medieval latrines generated a completely different pattern in terms of the “mass” occurrence of remains but also in size, most of the fish smaller than 10 cm (Fig. 8 above). The size distribution of common carp suggests a size transformation over time as well. Common carp from the Roman contexts widely ranged from about 20 cm up to very large specimens exceeding 1 m in length (Fig. 9). Medieval individuals indicated a shift towards individual lengths of 30–50 cm. Finally, the late/post-medieval finds implied size reduction and standardization at balanced median values of 40 cm (Fig. 9).

### Comparison of archaeological remains and written historical documents

The written sources confirm the fish species detected in the archaeological records but also complement and complete the ichthyo-faunal evidence. Besides sterlet and beluga sturgeon, Schmelzl (1547) additionally described the availability of waxdick and stellate sturgeon at the fish market in Vienna. Cyprinids such as silver bream, blue bream, dace, vimba bream or schneider were not explicitly mentioned in Galgóczi’s cookbook (1622) and were most probably summarized as “Weißfisch” in Schmelzl’s list (Table 2). Other cyprinids were commented in the cookbook, including barbel, common carp, common bream, bleak, asp, nase, ide, rudd or tench. Among percids, only zingel occurred neither in the Austrian nor in the Hungarian archaeological records. Schraetzer and ruffe appeared in the list of the Viennese fish market as well as in the archaeological record. Recipes for small fish such as loaches, bullhead but also burbot indicate exploitation in post-medieval Hungary. Other species absent from the Hungarian archaeological records, such as grayling, trout and Danube salmon, were again documented in the form of recipes only. Whitefish and alpine charr are mentioned in Schmelzl’s (1547) list for the Viennese fish market but lack accompanying archaeological proof as yet.

**Table 1:** Number of identified fish remains from the Austrian and Hungarian sites

	Austria tributary prehistory	Austria tributary medieval	Austria tributary late/post medieval	Austria Danube Roman	Austria Danube medieval	Austria Danube late/post medieval	Hungary upstream section prehistory
Pisces	185	1868	683	308	121	4493	279
Acipenserinae		19	27	18		9	7
*Acipenser ruthenus*					1		
*Huso huso*		16	4	1	6	1	3
*Anguilla anguilla*			22			31	
Cyprinidae	98	295	440	336	34	1527	242
*Ballerus ballerus*			1				
*Abramis brama*		1	2	3	1	1	16
*Alburnoides bipuncatatus*						1	
*Alburnus alburnus*				1		7	
*Aspius aspius*				17			
*Barbus barbus*		2	17	8	3	16	8
*Blicca bjoerkna*			1	2		4	1
*Carassius carassius*		1		1		1	5
*Chondrostoma nasus*			2	1		7	
*Cyprinus carpio*		317	309	168	16	65	443
Gobio sp.			8			19	
Leuciscus sp.	1	2	72	2		72	
*Leuciscus cephalus*	1	3	45				
*Leuciscus idus*			1	2			15
*Leuciscus leuciscus*			2				
*Leuciscus souffia*						1	
*Pelecus cultratus*							1
*Phoxinus phoxinus*			25			12	
*Rutilus rutlius*/sp.		1	9	9		79	15
*Scardinius erythrophthalmus*		2	5	1		3	
*Tinca tinca*			3	1			14
*Vimba vimba*							1
Cobitidae/Balitoridae			20	1		277	
*Silurus glanis*			3	35	2	3	44
*Esox lucius*		328	158	128	10	324	82
*Thymallus thymallus*		3	1				
*Hucho hucho*		1		2	1		
*Salmo trutta f. fario*	17	120	204	3		43	
*Lota lota*	1	7		2		19	
*Cottus gobio*		22	548			471	
Percidae	4	5		8		480	
*Sander lucioperca*		2		54		1	17
*Perca fluviatilis*	27	5	3	10	2	75	1
*Gymnocephalus cernua/baloni*						103	
*Gymnocephalus schraetser*						72	
Gadus sp.			85			1	
Scomberidae				17			
*Clupea harengus*/sp.		1	132	1		131	
Pleuronectiformes			6				
*Pleuronectes platessa*			60		1		
*Psetta maxima*			5				
Soleidae			2				
Marine fish				2			

	Hungary upstream section Roman	Hungary upstream section medieval	Hungary upstream section late/post medieval	Hungary downstream section prehistory	Hungary downstream section Roman	Hungary downstream section medieval	Hungary downstream section late/post medieval
Pisces	63	23	273	38	7	14	53
Acipenserinae	2	2	6	1	3	2	9
*Acipenser ruthenus*		10	3			5	28
*Huso huso*	1	25		1		3	4
*Anguilla anguilla*							
Cyprinidae	7	4		1		1	41
*Ballerus ballerus*							
*Abramis brama*							6
*Alburnoides bipuncatatus*							
*Alburnus alburnus*							
*Aspius aspius*							
*Barbus barbus*							
*Blicca bjoerkna*							
*Carassius carassius*							
*Chondrostoma nasus*							
*Cyprinus carpio*	10	47	15	170	1	20	63
Gobio sp.							
Leuciscus sp.							
*Leuciscus cephalus*							
*Leuciscus idus*							
*Leuciscus leuciscus*							
*Leuciscus souffia*							
*Pelecus cultratus*							
*Phoxinus phoxinus*							
*Rutilus rutlius*/sp.							
*Scardinius erythrophthalmus*							
*Tinca tinca*							
*Vimba vimba*							
Cobitidae/Balitoridae							
*Silurus glanis*		3	12			6	9
*Esox lucius*	9	8	5			5	45
*Thymallus thymallus*							
*Hucho hucho*							
*Salmo trutta f. fario*							
*Lota lota*							
*Cottus gobio*							
Percidae							
*Sander lucioperca*	2	1	2		2		2
*Perca fluviatilis*							
*Gymnocephalus cernua/baloni*							
*Gymnocephalus schraetser*							
Gadus sp.							
Scomberidae							
*Clupea harengus*/sp.							
Pleuronectiformes							
*Pleuronectes platessa*							
*Psetta maxima*							
Soleidae							
Marine fish

**Table 2:** List of fish species mentioned in manuscript of Galgóczi’s cookbook in 1622 and fish listed by Schmelzl (1547) available at the Viennese fish market in comparison to the late/post-medieval archaeoichthyological distribution in Hungary and Austria

		Galgóczi’s cookbook 1622 number of recipes	Late/post medieval Hungary	Schmelzl (1547)	Late/post medieval Austria
Lamprey	Eudontomyzon sp.	3		Neunaugen	
Sturgeon	Acipenser sp.	12	X		X
Sterlet	*Acipenser ruthenus*	11	X	Stierl	X
Waxdick	*Acipenser güldenstedti*			Tück	
Stellate sturgeon	*Acipenser stellatus*			Schierken	
Beluga sturgeon	*Huso huso*	12	X	Hausen	X
Eel	*Anguilla anguilla*	2		Aal	X
Gudgeon	Gobio sp.	1		Greßling	X
Barbel	Barbus sp.	3	X	Barben	X
Silver bream	*Blicca bjoerkna*		X		X
Common carp	*Cyprinus carpio*	19	X	Karpfen	X
Common carp	*Cyprinus carpio*			Seekarpfen, Theißkarpfen	
Crucian carp	*Carassius carassius*	8	X	Garauß	X
Bream	*Abramis brama*	1	X	Brachsen	X
Blue bream	*Ballerus ballerus*				X
Bleak	*Alburnus alburnus*	1			X
Asp	*Aspius aspius*	1			X
Nase	*Chondrostoma nasus*	1		Näsling, Kräuterling	X
Ide	*Leuciscus idus*	1	X	Nerfling	X
Chub	*Leuciscus cephalus*			Aelten	X
Dace	*Leuciscus leuciscus*				X
Roach	*Rutilus rutilus*		X	Rothäugl	X
Rudd	Scardinius erytrophthalmus	1			X
Tench	Tinca tinca	6	X		X
Ziege	Pelecus cultratus		X	Sichling	
Minnow	Phoxinus phoxinus			Elritze, Pfrillen	X
Vimba bream	Vimba vimba		X		
Schneider	Alburnoides bipuncatatus				X
Spined loach	Cobitis sp.			Steinbeiß	X
Weather loach	*Misgurnus fossilis*	13		Bißgurre	
Stone loach	*Barbatula barbatula*	4		Grundel	X
Sheatfish	*Silurus glanis*	9	X	Scheiden	X
Pike	*Esox lucius*	22	X	Hecht	X
Whitefish	Coregonus sp.			Reinanken	
Alpine charr	*Salvelinus alpinus*			Seibling	
Trout	*Salmo trutta f. fario*	8		Forellen	X
Danube salmon	*Hucho hucho*	6		Huchen	X
Atlantik salmon	*Salmo salar*	1			
Grayling	*Thymallus thymallus*	3		Aesche, Mailing, Sprenzling	X
Burbot	*Lota lota*	10		Rotten?	X
Bullhead	*Cottus gobio*	3		Koppen	X
Perch	*Perca fluviatilis*	2	X	Barsche	X
Pikeperch	*Sander lucioperca*	1	X	Schille	X
Zingel	*Zingel zingel*	1		Zindel?	
Schraetser	*Gymnocephalus schraetser*	1			X
Ruffe	*Gymnocephalus cernua*			Wachsfisch	X

## Discussion

Archaeological fish remains produce distorted pictures that are biased by taphonomic history, beginning with fishing, trade and consumption and ending with preservation. Beyond methodological factors such as the expertise or the method of recovery, which massively influence the richness of species by “sampling” efforts, the state of preservation and inconsistent quantities of identifiable bones impedes chronological and contextual comparisons. The proxies provide a reliable signal at least for the regularly hand-collected large fish species, bearing in mind that limited material may reflect regional—rather than diachronic variations.

However, as a recurring nutritive resource, large migratory sturgeons were targeted from prehistoric times on at the Hungarian part of the Danube. The first Austrian sturgeon remains date to the Roman period, while the prehistoric site Ansfelden is located outside their natural migration routes and no evidence of prehistoric sturgeon exploitation has been proven to date along Austria’s Danube. Besides the archaeologically evidenced sterlet and beluga sturgeon, a higher variety of sturgeon species is expectable (Schmelzl 1547; Zauner 1997). The Austrian Danube offered good sturgeon fishing grounds (Balon 1968) and, since medieval times, sturgeon fishing intensified (Bartosiewicz and Bonsall 2008). As opposed to Hungary, the assumption is that sturgeon populations in Austria already declined in Early Modern times, which may explain their disappearance from fishing regulations in the 16th century (Haidvogl et al. 2013). Especially the economically and symbolically important beluga sturgeons were continuously delivered from Hungary to the Viennese fish market (Reischl 1921/1922) and even transported to sites at small Danube tributaries (Galik and Kunst 2000). As indicated by archaeoichthyological remains excessive fishing finally reduced the size and abundance of long-distance migratory sturgeons along the Hungarian part as well (Balon 1968; Bartosiewicz et al. 2008; Guti 2008).

The remains from the Roman latrine in the Vienna Basin indicate another pattern as the late/post-medieval latrine fillings. The Roman site reveals the use of only a few small sized fishes although the sediment was carefully sieved. Even smaller sizes and intensified exploitation of juvenile cyprinids and other small species indicate changes in fisheries in the late/post-medieval times in Austria. Historical records refer to these environmental impacts, in particular fishing laws such as introduced by Albrecht V in 1412 for the Austrian part of the Danube (Uhlirz 1900). They prohibited the use of specific nets to protect purposefully juvenile individuals of larger species as well as other small fishes. Although not present in Hungarian sites, small species such as bullhead, loaches as well as lamprey represented popular delicacies as indicated by Galgóczi’s (1622) cookbook. Comparable other sites with latrines in Switzerland and Germany probably raise this phenomenon on specific exploitation of small and young fish to Central European behaviour at this specific period (Heinrich 1995; Brombacher et al. 1998; Hüster-Plogmann 2006; Nussbaumer and Rehazek 2007).

Common carp and other cyprinids and pike are abundant and important fish in Austria and Hungary and probably mirror an increase of fishing intensity towards the medieval- but a decrease of specimens towards the late/post-medieval periods. The Austrian common carp remains reflect human impact at the ecosystem, too. Comparable to Hungarian Neolithic exploitation (Bartosiewicz 2013) the Austrian Roman contexts demonstrate exploitation of the Danube’s wild carp populations, revealing a wide range of individual size. Common carp was stocked in ponds since the early medieval period (Hoffmann 1994, 1995, 1996, 2005; Balon 1995) and massively reared in the High Middle Ages. The estimated number of man-made Hungarian fishponds ranges from 3000 to 4000 from the 11th to the 13th century (Pesty 1867). Loads of harvested reared common carps of similar size and weight suggest a compensation of declining natural wild carp stocks; this is underlined by the “size standardization” at about 40 cm individual lengths in medieval and late/post-medieval contexts. Schmelzl (1547) differentiates between certain forms of common carp “*Karpfen, Seekarpfen*” and “*Theißkarpfen”* most probably from the Tisza River. Besides these forms, Bohemian reared common carp was transported to the Viennese fish market as well (Wacha 1956).

The documentation of assumed ecological differences and changing fish communities between the Austrian and the Hungarian part of the Danube is hampered by the aforementioned methodological insufficiencies and is restricted to mainly larger eurytopic species. Even such hand-collected and large fish remains indicate that species such as sturgeon, common carp or pike had been transported to remote sites at Austria’s Danube tributaries, outside their natural habitat. At a finer level, the sieved samples reveal a correspondence of species composition and Danube habitats in the Vienna Basin and the tributary sites. The conspicuous absence of trout in early medieval Ansfelden might be explained as a pattern of Slavic people’s preference for cyprinids, whereby potential taphonomic conditions or environmental changes may also have played a role. Typical species in the samples such as schraetzer and ruffe indicate exploitation of local Danube fish in the Vienna Basin (Zauner 1996). A good representation of large remains of pikeperch is expected because they are more easily recognisable even by hand collection. They accumulated in Roman time but prove to be sporadic in later periods. Such a pattern indicated by these archaeoichthyological remains may relate to environmental changes as pikeperch requires aerated waters. Even subtle shifts in temperature or velocity (Bartosiewicz and Bonsall 2004) impact the post-Roman Danube habitats in Austria and Hungary, although without historical habitat information this assumption is difficult to prove. Nevertheless Galgóczi’s (1622) cookbook mentions only a single pikeperch recipe, at least pointing to a lesser culinary interest in the 17th century. The absence of grayling and salmonids in the Hungarian part of the Danube might be argued by temperature thresholds and fewer suitable habitats and these species occurred mainly at sites along the Danube tributaries even in Austria. Nevertheless, the Hungarian cookbook comprises numerous “salmonid”-recipes, indicating a 17th century supply of these species from cold-water tributaries or, more likely, fish trade. Austrian historical sources reveal brown trout and alpine charr as fresh deliveries to the Viennese fish market in the 16th century (Wacha 1956). Based on our samples the absence of burbot in Hungary is contradicted by ten recipes in the cookbook, underlining the popularity of this fish in Hungarian gastronomy.

Imported and preserved marine fish certainly reflect a demand for Mediterranean cuisine and life style in land-locked Central Europe in Roman times (Hüster-Plogmann 2002; Galik 2004; Van Neer and Ervynck 2004; Hüster-Plogmann 2006; Galik et al. 2009). Most of the imported fish was certainly of Mediterranean origin, but a Roman pit filling in Vienna at the “*Freyung*” yielded, beyond few Atlantic chub mackerel remains, vertebrae resembling small-sized herring and strongly indicating the import of Atlantic fishes. The late medieval eel remains may represent preserved and transported fish as well, but historical records show that eel migrated towards two Austrian regions. The first was the Lainsitz in Lower Austria, where eel entered from the Elbe-system. The second was Lake Constance via the Rhine River (Spindler 1997). Nonetheless, the massive medieval and post-medieval import of marine fish (Lampen 2000; Locker 2001)—even to deep inland areas (Zeiringer 1991) associated with increasing fish farming—suggests a compensation for an under-supply of resources with cheap salted fish as mentioned in the “Fisch Preis Taxe in Eger” in 1465 (Abel 1980). Although there is no archaeological evidence for such fish remains in Hungary, several recipes were prepared using marine fish in the incomplete manuscript of Fay’s Hungarian cookbook from the 17th century (Herman 1887).

Concluding, our investigations demonstrated the potential of using fish remains and written historical records for reconstructing long-term changes of the Danube´s fish communities but also certain limitations as well as the dependence from the methodological approaches e.g. of sampling. Although only proxy data are involved, the species found and the frequency of bones clearly indicate the relation between the fish community and human interaction and impact on the Danube’s environment. Our results strongly encourage improvement and standardization of archaeological recovery methods and routine screening for fish remains. Such data can provide historical investigations at finer chronological and geographical resolutions along the various riverine habitats of the Danube. They have the potential to enlighten even local changes in ecology and fish population. The incorporation of new and well established bio-molecular methods with still rising potential and reliability such as analyses of stable isotopes (Barrett et al. 2004, 2011; Orton et al. 2011; Fuller et al. 2012) and aDNA (Hlinka et al. 2002; Arndt et al. 2003) can improve the identification of fish species and elicit the provenance of traded fish. Most promising for the future will be the combination of methods such as morphology, osteometry, isotopes and aDNA (Ólafsdóttir et al. 2014) even for the development of fresh water fish populations along the Danube.
